# A Combined Approach to Predicting Rest in Dogs Using Accelerometers

**DOI:** 10.3390/s18082649

**Published:** 2018-08-13

**Authors:** Cassim Ladha, Christy L. Hoffman

**Affiliations:** 1 VetSens, 53 Wellburn Park, Newcastle NE2 2JY, UK; cas@vetsens.co.uk; 2 Department of Animal Behavior, Ecology, and Conservation, Canisius College, Buffalo, NY 14208, USA

**Keywords:** dog, activity recognition, actigraphy, rest, behavior

## Abstract

The ability to objectively measure episodes of rest has clear application for assessing health and well-being. Accelerometers afford a sensitive platform for doing so and have demonstrated their use in many human-based trials and interventions. Current state of the art methods for predicting sleep from accelerometer signals are either based on posture or low movement. While both have proven to be sensitive in humans, the methods do not directly transfer well to dogs, possibly because dogs are commonly alert but physically inactive when recumbent. In this paper, we combine a previously validated low-movement algorithm developed for humans and a posture-based algorithm developed for dogs. The hybrid approach was tested on 12 healthy dogs of varying breeds and sizes in their homes. The approach predicted state of rest with a mean accuracy of 0.86 (SD = 0.08). Furthermore, when a dog was in a resting state, the method was able to distinguish between head up and head down posture with a mean accuracy of 0.90 (SD = 0.08). This approach can be applied in a variety of contexts to assess how factors, such as changes in housing conditions or medication, may influence a dog’s resting patterns.

## 1. Introduction

Actigraphy can provide a noninvasive way to objectively measure episodes of rest and thereby provide information about an individual’s health and well-being. Studies in humans have used actigraphy-driven sleep measurement techniques, such as the Estimation of Stationary Sleep-Segments (ESS) approach described by Borazio et al. [1], to test the effectiveness of medications and medical treatments [2], and to identify individuals with heightened susceptibility to disease, cognitive impairment, and/or mortality [3,4,5]. Actigraphy-based studies of rest patterns in dogs may provide similar insights into dog health and well-being, but differences in dog and human postures and movement patterns necessitate the development of dog-specific algorithms related to resting behavior. When a human is lying down, the individual is likely to be sleeping, whereas they are more likely to be resting but not sleeping when sitting. Dogs, on the other hand, are not necessarily in a sleeping state when they are lying down, as they are commonly observed in a recumbent position with their head elevated and supported by their neck muscles. For this reason, posture-based approaches, such as one developed by Lugade et al. for estimating human resting behaviour [6], are not well-suited for assessing dog resting behaviour. Instead, the most accurate measures of dog resting behaviour may require information about both movement intensity and head position. An accurate method for identifying truly relaxed behaviours is needed to characterize dogs’ typical activity patterns and deviations that may be indicative of health- or welfare-related problems.

This paper is comprised of six sections. Section 2 describes prior actigraphy-based studies that have investigated resting behaviour in dogs and provides the aims of the current study; Section 3 details our study methods and analysis process; Section 4 reports on and compares the performance of the three approaches we used to assess resting behaviour in dogs; Section 5 discusses the strengths and weaknesses of each approach and the potential applications for the novel approach presented in this paper; and Section 6 highlights the conclusions that can be drawn from this investigation.

## 2. Background and Motivation

Actigraphy-based studies of dogs have successfully identified physical activity levels and even specific behaviours [7,8,9], and a few prior studies have used actigraphy to assess sleep or rest in dogs [10,11,12,13]. John et al. examined movement data gathered from accelerometers placed on a cohort of laboratory-housed dogs [10]. Data were summarised in 5-min epochs and compared against polysomnography results. Findings from the actigraphy and polysomnography measures showed convergence, and the same actigraphy-based method was employed successfully in two additional studies of rest and activity patterns in laboratory-housed dogs [11,12]. The accuracy of the method used in these studies would be questionable across dogs of varying sizes and breeds and outside the laboratory environment, however. All three of these studies relied on a single axis accelerometer, which is prone to rotation errors when the dog’s collar shifts and so may not perform as reliably in a home environment where dogs move freely. The extent of the collar rotation error problem is quantified by Olsen et al. [14], which makes recommendations to standardise collar tightness; however, standardization is not necessarily practical.

Clarke and Fraser devised a method for measuring rest that extends to dogs kept as companions [13]. They combined filming and actigraphy of owned dogs and shelter dogs confined in exercise pens to determine how well an accelerometer identified periods of rest. The authors found that head posture predicted periods of rest reasonably well; however, the method did not properly identify dogs as resting when they were in recumbent positions but with their heads on an elevated substrate. This algorithm may also fail if the collar rotates or if dogs are resting on their sides.

**Figure 1:**
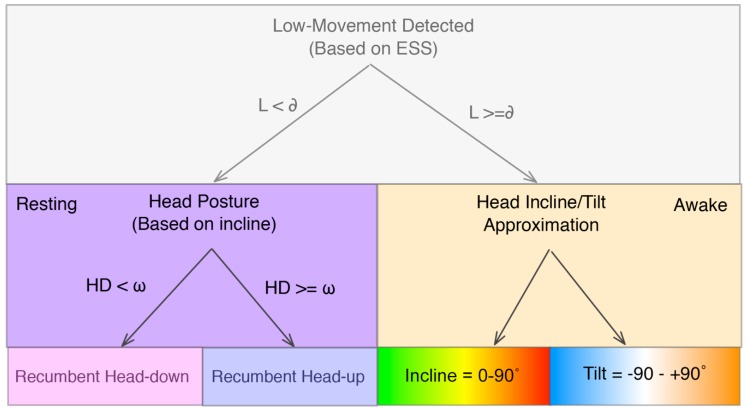
State diagram of our proposed LMR approach.

The current study aimed to produce a novel algorithm that can successfully predict whether a dog is alert or resting when inactive. We combined Clarke and Fraser’s [13] method with the ESS approach, which is a validated method for identifying human sleep using low movement detection [1]. The proposed approach also makes use of a rotation correction step designed to re-orient sensor data as though it originated from an under-chin sensor position [15]. We started by tailoring the low-movement detector so that it would be sensitive to dogs. This allowed for the classification of resting and non-resting states. Subsequently, we applied Clarke and Fraser’s algorithm in isolation on our dataset and compared performance to our low-movement detection algorithm. Finally, we combined the approaches, creating the Low Movement Recumbency (LMR) approach, and evaluated the resulting algorithm’s performance in a naturalistic setting. Our proposed approach is shown in Figure 1. We hypothesized that this hybrid approach would succeed at discriminating between when dogs were resting with their head up versus down. In short, we used the ESS approach to identify low movement and thus discriminate between Rest and Active states. Within the Rest state, we applied Clarke and Fraser’s algorithm to distinguish Recumbent-Head Down from Recumbent-Head Up states.

## 3. Materials and Methods

### 3.1. Animals

The study involved a convenience sample of 12 owned dogs living within 20 km of Buffalo, NY, USA. The owners of these dogs reported that their dogs were in good health, and none displayed any locomotor abnormalities. Dogs ranged in age from 3 to 13 years and in size from 7 to 31 kg. Table 1 provides a more detailed description of the dogs. Canisius College’s Institutional Animal Care and Use Committee approved the study prior to the onset of data collection (protocol number 2017-431).

### 3.2. Collection Protocol

Data were collected in participants’ homes between the hours of 0800 and 1800 during June 2017. Each dog’s owner indicated where their dog tended to spend the most time resting during daytime hours, and we set up a GoPro Hero 3+ camera (GoPro Inc., San Mateo, CA, USA) in that location to continuously record the dog’s activity. We mounted the GoPro onto a tripod to capture as much of the room as possible.

Each dog wore a tri-axial accelerometer sensor (VetSens, Newcastle, UK). The sensors were configured to sample acceleration (*f_s_*) at 100 Hz (100 samples in each axis per second), as this frequency was previously shown to be suitable for capturing canine movement [7]. Sensors were attached ventrally to each dog’s collar using Gorilla Tape (Gorilla Glue Company, Cincinnati, OH, USA). Before attachment to the subject, the instrumented collar was held in front of the camera while clapping five times. At the end of the data collection period, the collar was removed from the dog and once again held in front of the camera while clapping five times. The clapping procedure ensured a recognisable trace in the data stream that could subsequently be used for alignment of video and actigraphy data. Author 2 remained in two dogs’ homes for the entire data collection period but not in the same room as the dog. For four of the dogs, Author 2 remained in the room with the dog during data collection but ignored the dog to minimize her impact on the dog’s behaviour. For the other six dogs, Author 2 set up and retrieved the recording equipment but was not present during the data collection period.

### 3.3. Data Analysis

Tri-axial accelerometer data from each dog was subjected to a rotational correction algorithm described in Ladha et al. [15]. This approach removes the effect of the collar not being positioned precisely ventrally or rotating out of position. The resulting data have the *z*-axis of the accelerometer aligned with the dorsal-ventral (*DV*) axis of the subject. The data from each subject was then processed with two algorithms: the ESS approach detailed by Borazio et al. [1] and the head-down estimator detailed by Clarke and Fraser [13].

The ESS approach starts by discarding the anterior-posterior (AP) and medial-lateral (ML) data and subjecting the remaining *DV* data to a strong low-pass filter (1st order Infinite Impulse Response (IIR) of Butterworth design. *f_c_* = 3 Hz and −60 db at 11 Hz) [1]. The filtered data are then split into non-overlapping, 1 s windows. For each window, the standard deviation is calculated, and low movement is predicted when the standard deviation is below a threshold (*δ*). Formally, this can be expressed as:

(1) $$L_{\delta }=\{\begin{array}{l} 1,\;if\;\sqrt{\frac{1}{100}\overset{100}{\underset{i=1}{\sum }}{\left(DV_{i}-\bar{DV}\right)}^{2}}>\delta \\ 0,\;otherwise \end{array}$$

Here, *L* is predicted low movement and remembering that *f_s_* = 100 Hz.

Clarke and Fraser’s [13] method for predicting head-down recumbency works by thresholding the head-incline angle over a period of time. We chose to replicate this method as a baseline for evaluating our proposed enhancements. Clarke and Fraser did not describe in technical detail how they approximated head incline from the raw accelerometer data. In our implementation, pitch and roll from the tri-axial accelerometer can be done using the following expressions:

(2) $$\tan\theta_{xyz}=\frac{-A_{x}}{\sqrt{A_{y}{}^{2}+A_{z}{}^{2}}}$$

(3) $$\tan\phi_{xyz}=\frac{A_{y}}{\mathrm{sign}(A_{z})+\sqrt{A_{z}{}^{2}+\mu A_{x}{}^{2}}}$$

Here *θ* and *ϕ* denote the pitch and roll angles, respectively. *μ* is a modifier to improve stability in the case where the denominator of Equation (3) becomes small (value fixed at 0.1). The *xyz* subscript represents the angles, which describe rotation with the aerospace convention such that any rotation can be described by the rotation sequence ***R****_xyz_* as:

(4) $$R_{xyz}=\left(\begin{array}{c} -\sin\theta \\ \cos\theta \sin\phi \\ \cos\theta \cos\phi \end{array}\right)$$

**Figure 2:**
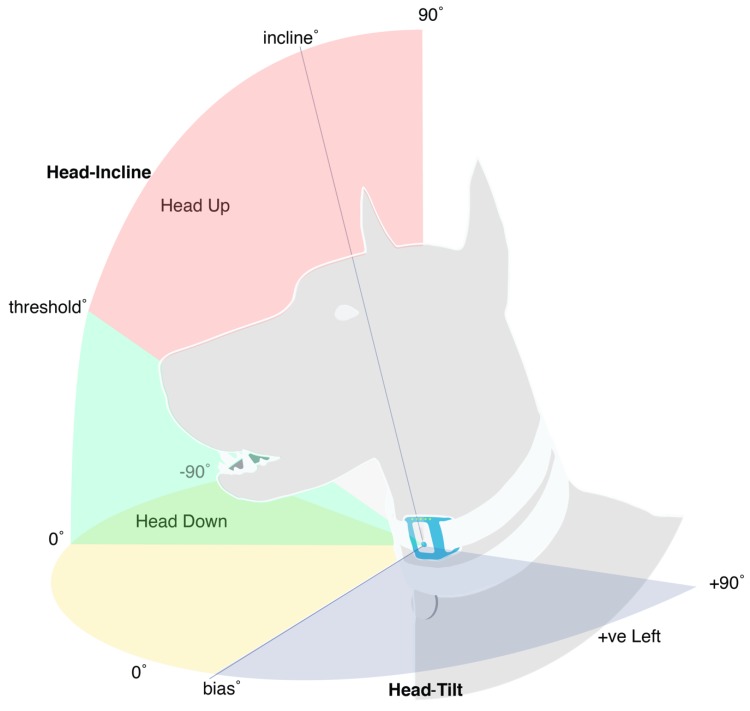
Pitch and Roll angles of the sensor map to head-tilt and head incline, respectively. The angle of 14° was found to be the optimised threshold to classify head-down posture while recumbent.

With the VetSens sensors, pitch maps to an incline (“head-tilt”) angle orthonormal to the cranial-caudal axis of the dog (positive tilting left from the dog’s perspective). Roll maps to an incline (“head-incline”) angle orthonormal to the medial-lateral axis of the dog (positive tilting the head away from the floor). Figure 2 describes these variables pictorially.

As with Clarke and Fraser’s algorithm [13], a threshold was set on the mean incline angle over a window of 10 s width. This approach is designed to mitigate rapid movements such as headshaking. Formally, the head-down prediction can be written as:

(5) $$HD_{\tau }=\{\begin{array}{l} 1,\;if\;\frac{\sum_{i=0}^{999}\theta }{1000}\geq \omega \\ 0,\;otherwise \end{array}$$

In this formula, *HD* is the predicted head incline angle over the 10 s window (remembering that *f_s_* = 100 Hz), and *ω* is the threshold angle on Head-Incline angle.

### 3.4. Annotations

Videos were annotated for Out of View, Rest, Rest-Head Up, and Not Resting using the ethogram provided in Table 2 and ELAN 5.0.0 software (Max Planck Institute, Nijmegen, The Netherlands). Of note, “Rest-Head Up” occurred when the dog was not moving but the head was supported by the dog’s neck muscles. Periods where the dog was resting but not supporting his own head would be interpreted as “Rest”, even if the head was on an elevated surface that put the head out of line with the rest of the dog’s body.

From the annotations, periods of Recumbent Head-Down Rest were extracted as periods that were annotated as Rest and not Rest-Head Up. Periods of Alert were taken as periods that were not annotated as Rest. All sections of video annotated Out of View were excluded from the analysis.

The claps inserted into the data stream were identified using manual inspection, and time offsets that enabled synchronisation were calculated. One individual annotated all twelve videos, and a second individual annotated four of the videos to confirm inter-observer reliability. Inter-observer reliability was tested in the ELAN software using the algorithm developed by Holle and Rein [16] and an overlap criterion of 60%. Cohen’s Kappa was 0.83.

### 3.5. Statistics

To determine the performance of our algorithm, the following definitions were made:

- True Positives (*TP*) were defined as time periods where the output prediction and the annotations agreed on a positive state such as Rest or Head Up.
- False Positives (*FP*) were defined as time periods where the output prediction specified a positive state but the annotations did not agree.
- True Negatives (*TN*) were defined as time periods where the prediction and the annotations agreed on a negative state such as Not Resting or Not Head Up.
- False Negatives (*FN*) were defined as time periods where the output prediction specified a negative state but the annotations did not agree.

States to predict were defined as Low-Movement and subject in a Head-Down posture. Positive Predictive Value (*PPV*), Negative Predictive Value (*NPV*), Specificity (*SPC*), Sensitivity, (*SEN*) and Accuracy (*ACC*) were then calculated as:

(6) $$PPV=\frac{TP}{TP+FP}$$

(7) $$NPV=\frac{TN}{TN+FN}$$

(8) $$SPC=\frac{TN}{TN+FP}$$

(9) $$SEN=\frac{TP}{TP+FN}$$

(10) $$ACC=\frac{TP+FP}{TP+FP+FN+TN}$$

### 3.6. Determining the Best-Fit Thresholds

To obtain generalised thresholds *δ* and *ω* that could be potentially carried forwards and used on any dog, we first did a subject-by-subject optimization. In this exercise, each threshold was varied and resulting sensitivity and specificity recorded. An optimum value was described by giving the best compromise between sensitivity and specificity (a crossing point on the receiver operating characteristic, ROC, curve). Once tuned thresholds were obtained for each dog, group-based values were established as the best fit solution.

## 4. Results

All participants were accustomed to wearing collars, and none showed any behavioural indicators that the collar or sensor impacted them. The sensors and recording equipment functioned properly for all 12 dogs. Recording sessions ranged in length from 99 min to 205 min (average recording length: 144 min). Exact details of recording length for each dog are in Table 1, as are the times spent in a resting state according to annotations.

### 4.1. Predicting Rest from Low Movement (ESS Approach)

**Figure 3:**
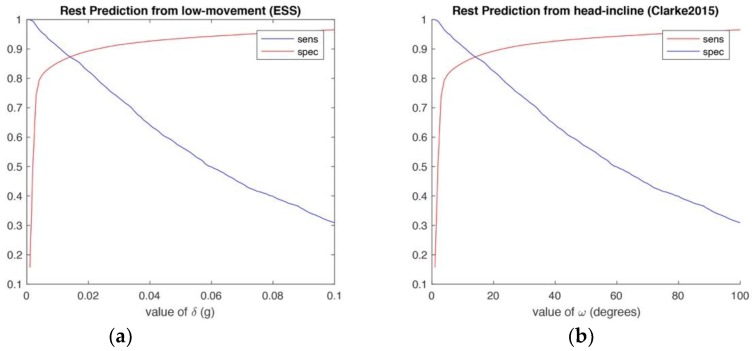
(**a**) ROC approach to find the optimal movement threshold of *δ* for rest and non-rest classification using the ESS approach; (**b**) ROC approach to determine optimal angle of *ω* for classification of head-up vs. head-down while recumbent as in Clarke and Fraser.

The ESS algorithm was subject to a best-fit approach to determine the generalised threshold value for all dogs. It was determined a value of *δ* = 0.014 g gave the optimal sensitivity and specificity over our dataset (see Figure 3a). This value produced a mean accuracy of 0.86 (SD = 0.08). The performance did not seem to favour or discriminate against any individual subject. Results for each subject are shown in Table 1.

### 4.2. Head Down Rest

To determine the best generalised head-incline angle that predicted head-up and head-down position, a second best-fit experiment was run. This yielded a cut-off angle *ω* = 14° (see Figure 3b) that performed with a mean accuracy of 0.89 (SD = 0.06). This is only slightly different from the optimal angle found in Clarke and Fraser [13], which reported a mean accuracy of 0.87 for an angle of 10°. In our dataset, the algorithm performed consistently for all but one subject (Bruno), who had a prediction accuracy of 0.78.

### 4.3. LMR Approach

The LMR method was able to classify the Recumbent-Head Up state with a mean accuracy of 0.90 (SD = 0.08). The performance over all subjects was consistent, and the lowest accuracy (0.74) was observed in the same dog (Bruno) for which the Recumbent Head-Up detection performed the most poorly.

In terms of ability to reject false predictions, the specificity and negative predictive value (NPV) for each dog is also reported (see Table 1), and mean values were 0.84 and 0.94, respectively. This result is important because if the predictions from the algorithm are to be relied upon, then there must be confidence in their correctness.

We attempted to correlate *ω* and *δ* with height and weight but were unable to find a significant relationship. Thus, there is no opportunity to tailor the LMR approach to an individual based on these parameters.

## 5. Discussion

The LMR approach functions well and utilizes features from Clarke and Fraser’s approach [13] and the ESS approach [1]. It discriminates rest from active states but has the added value of distinguishing between when the dog is resting with the head in line with the body and when the dog is resting with the head elevated in relation to the body. We hypothesize that a dog who is recumbent and has their head in line with their body (i.e., down) is more likely to be asleep, or at least in a relaxed state, whereas a dog recumbent with head up is more likely to be in an alert state.

**Figure 4:**
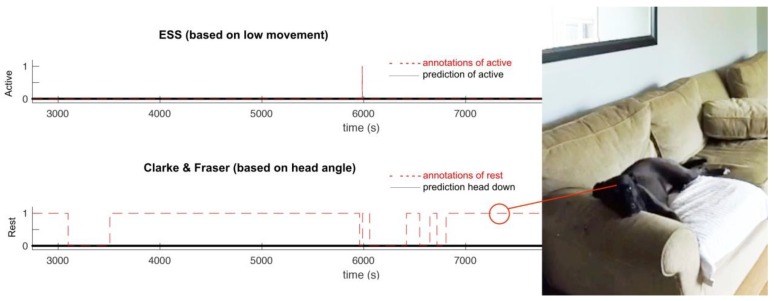
The ESS approach succeeds at predicting Rest (as not active) on low movement detection (prediction matches annotation). Clarke and Fraser’s method fails to predict Rest based on head angle. This case highlights problems predicting Rest based on head angle alone.

For the one dog who had lower performance using Clarke and Fraser’s approach [13], we investigated where the algorithm was failing to classify states properly. In this particular instance, the dog had chosen to rest on a couch with his head supported on the arm-rest. The dog was annotated as being in a state of Rest, rather than in a state of Rest-Head Up, because his neck muscles were not supporting his head. This case highlights a shortcoming where if Clarke and Fraser’s approach [13] is used in isolation, the dog would not be properly classified as resting. In this particular example, the ESS approach properly classified the rest state because dog movement was below the cut-off threshold. Due to the decision tree nature of the LMR approach, a correct classification of rest was made, but of the algorithm misclassified the dog’s behaviour as Rest-Head Up since the ethogram stated the head must be supported by neck muscles to be in an up-position. This case highlights a possible area for future improvement for the LMR approach and illustrates that a dog’s head is not necessarily aligned with the dog’s body, even when it is fully supported by a substrate. To illustrate the above findings, Figure 4 is included and shows the prediction from each algorithm and a photo of the dog’s state.

The ESS approach created by Borazio et al. [1] has been validated in humans against polysomnography (PSG) to accurately predict sleep with a median precision of 79%. In our study, we found that on its own, the ESS approach had a mean accuracy of 0.86 for identifying dog resting behaviour. At this stage, without a PSG validation experiment, it is not possible to determine what proportion of detected rest in dogs would classify as sleep. However, our result is still promising because the ESS approach was devised for human use yet adapted well for use in dogs. Furthermore, the high accuracy of the ESS approach is important for the subsequent LMR approach. Due to the hierarchical tree associated with the LMR approach, a poor sensitivity in the movement-based stage would impact subsequent stages.

Of note, there was a slight difference in optimal incline angles between our approach and Clarke and Fraser’s [13], which could be due to slight sensor differences, the experiment environment, or the sample of dogs used in testing and validation. Additionally, in our experiment, we chose not to partition the dataset for developing and testing the optimal threshold angle. This decision creates the possibility of introducing an “over-fitting” solution; however, due to the sample numbers involved, it was deemed an appropriate approach. The difference between results may also be impacted by the LMR method correcting for rotational errors while Clarke and Fraser’s [13] method does not. The mean accuracy is very similar for both methods but does suggest that there should be a validation approach if different sensor designs are used.

The results indicate the LMR approach is sensitive enough to be used as a method for estimating rest, with added benefits over previous approaches that have utilized either movement or head position. The specificity and NPV of the LMR approach are also high, which is inspiring for applications wishing to use predictions for research purposes.

The extra level of insight from the LMR approach is anticipated to be useful when partitioning relaxed states from the Recumbent-Alert State (head-up) and may allow for more thorough assessments of dogs welfare in various contexts. For example, previous research based on observational methods suggests a positive relationship between daytime rest and well-being [17]. However, the behavioural methods employed in that study did not discriminate between the Recumbent-Alert State and the Recumbent Head-Down State, meaning it was not possible to differentiate periods of true restfulness from periods associated with vigilance or alertness. By distinguishing between different forms of rest behaviour, the LMR approach could offer an automatic classification mechanism and may serve as a more refined predictor of behavioural states and well-being.

## 6. Conclusions

The LMR approach combines methods devised by Clarke and Fraser [13] and Borazio et al. [1] and provides insights into dogs’ resting behaviours within a naturalistic environment. The performance suggests it is sensitive enough to be used as a rest detection method but with the added benefit of identifying head position. To determine whether resting with the head down is a strong predictor of the physiological state of sleep and to identify what connections can be drawn between a dog’s resting patterns and their health and welfare, further work is required. Nevertheless, even without additional validation, the method can be applied in a variety of contexts to assess how factors, such as changes in housing conditions or medications, may influence a dog’s resting patterns.

## Figures and Tables

**Table 1:** Description of study participants and performance of the ESS, Clarke and Fraser, and LMR methods.

Name	Breed	Age (years)	Weight (kg)	Height (cm)	Sex	Recording Duration (mins)	Time Resting (mins)	Accuracy of Rest Prediction Using ESS Only	Accuracy of Rest Prediction Using Clarke & Fraser	Accuracy of Recumbent Alert (LMR)	SPEC of Recumbent Alert (LMR)	NPV of Recumbent Alert (LMR)
Grizzly	Rottweiler Mix	10	31	56	F	202.70	185.96	0.87	0.89	0.92	0.96	0.90
Santiago	Pit Bull Mix	6	26	51	M	136.49	133.85	0.95	0.87	0.91	0.92	0.97
Molly	Shih Tzu	12	7	23	F	91.57	90.96	0.90	0.89	0.94	0.98	0.95
Cannoli	Cavalier King Charles/Bichon Frise	7	11	36	F	95.60	80.68	0.83	0.87	0.84	0.95	0.90
Oscar	Pug	13	16	31	M	126.30	124.86	0.91	0.97	0.91	0.92	0.96
Layla	Labrador Mix	4	25	48	F	194.76	184.88	0.91	0.91	0.85	0.66	0.97
Rico	Labrador Mix	4	25	56	M	198.43	181.93	0.90	0.86	0.78	0.69	0.97
Bruno	Labrador/Poodle	3	24	53	M	121.05	61.85	0.81	0.78	0.74	0.91	0.95
Ginger	Bulldog Mix	3	19	32	F	89.80	85.41	0.86	0.97	0.97	0.48	0.98
Charlie	Cavalier King Charles	11	13	39	M	90.82	89.42	0.78	0.93	0.99	0.98	0.97
Penny	English Toy Spaniel	6	9	28	F	75.74	73.91	0.65	0.84	0.95	0.86	0.87
Lulu	Terrier Mix	9	27	52	F	125.25	123.05	0.93	0.94	0.95	0.84	0.95

**Table 2:** Annotation Ethogram.

Rest	No movement of any body part with head not supported by neck muscles while lying down or sitting
Resting-Head Up	No movement of any body part with head supported by neck muscles while lying down or sitting
Not Resting	Standing or movement of the head or trunk that lasts for more than one second
Out of View (OOV)	Dog’s head and collar cannot be seen; at the beginning of the video, dog is scored as OOV until experimenter has placed collar and moved hands away from the dog; at the end of the video, dog is scored as OOV as soon as the experimenter’s hand touches the collar
